# 

**Published:** 1898-01

**Authors:** 


JANUARY, 1898.

LIBRARY
SURGEON GENERAL'S OFFICE
FEB.-5--1898

### THE AMERICAN JOURNAL OF DENTAL SCIENCE.

PUBLISHED MONTHLY.

$2.50 PER ANNUM.

EDITED BY

F. J. S. GORGAS, M. D., D. D. S.

RICHARD GRADY, M. D., D. D. S.

Vol. 31. THIRD SERIES No. 9.

BALTIMORE:

SNOWDEN & COWMAN MFG. CO., 9 W. FAYETTE ST.

LONDON:
TRUBNER & CO., 60 PATERNOSTER ROW.

Entered at the Post Office at Baltimore, Md., as Second-class Matter.

PAYABLE IN ADVANCE.

